# Concept and performance evaluation of two 3 GHz buncher units optimizing the dose rate of a novel preclinical proton minibeam irradiation facility

**DOI:** 10.1371/journal.pone.0258477

**Published:** 2021-10-11

**Authors:** Michael Mayerhofer, Andreas Bergmaier, Gerd Datzmann, Hermann Hagn, Ricardo Helm, Johannes Mitteneder, Ralf Schubert, Luigi Picardi, Paolo Nenzi, Concetta Ronsivalle, Hans-Friedrich Wirth, Günther Dollinger

**Affiliations:** 1 Universität der Bundeswehr München, Institut für Angewandte Physik und Messtechnik (LRT2), Neubiberg, Bavaria, Germany; 2 ENEA, Frascati, Rome, Italy; 3 Ludwig-Maximilians-Universität München, Fakultät für Physik, München, Bavaria, Germany; University of Seville, SPAIN

## Abstract

To demonstrate the large potential of proton minibeam radiotherapy (pMBRT) as a new method to treat tumor diseases, a preclinical proton minibeam radiation facility was designed. It is based on a tandem Van-de-Graaff accelerator providing a 16 MeV proton beam and a 3 GHz linac post-accelerator (designs: AVO-ADAM S.A, Geneva, Switzerland and ENEA, Frascati, Italy). To enhance the transmission of the tandem beam through the post-accelerator by a factor of 3, two drift tube buncher units were designed and constructed: A brazed 5-gap structure (adapted SCDTL tank of the TOP-IMPLART project (ENEA)) and a non-brazed low budget 4-gap structure. Both are made of copper. The performance of the two differently manufactured units was evaluated using a 16 MeV tandem accelerator beam and a Q3D magnetic spectrograph. Both buncher units achieve the required summed voltage amplitude of 42 kV and amplitude stability at a power feed of less than 800 W.

## Introduction

The number of new cancer cases is increasing worldwide (over 19 million in 2020) [1], at the same time, tumor-induced mortality was be further reduced by 5% on average in Europe between 2015 and 2020 [2]. Radiation therapy contributes to this trend in the majority of all cases and especially charged particle therapy becomes increasingly important [3].

**Fig 1:**
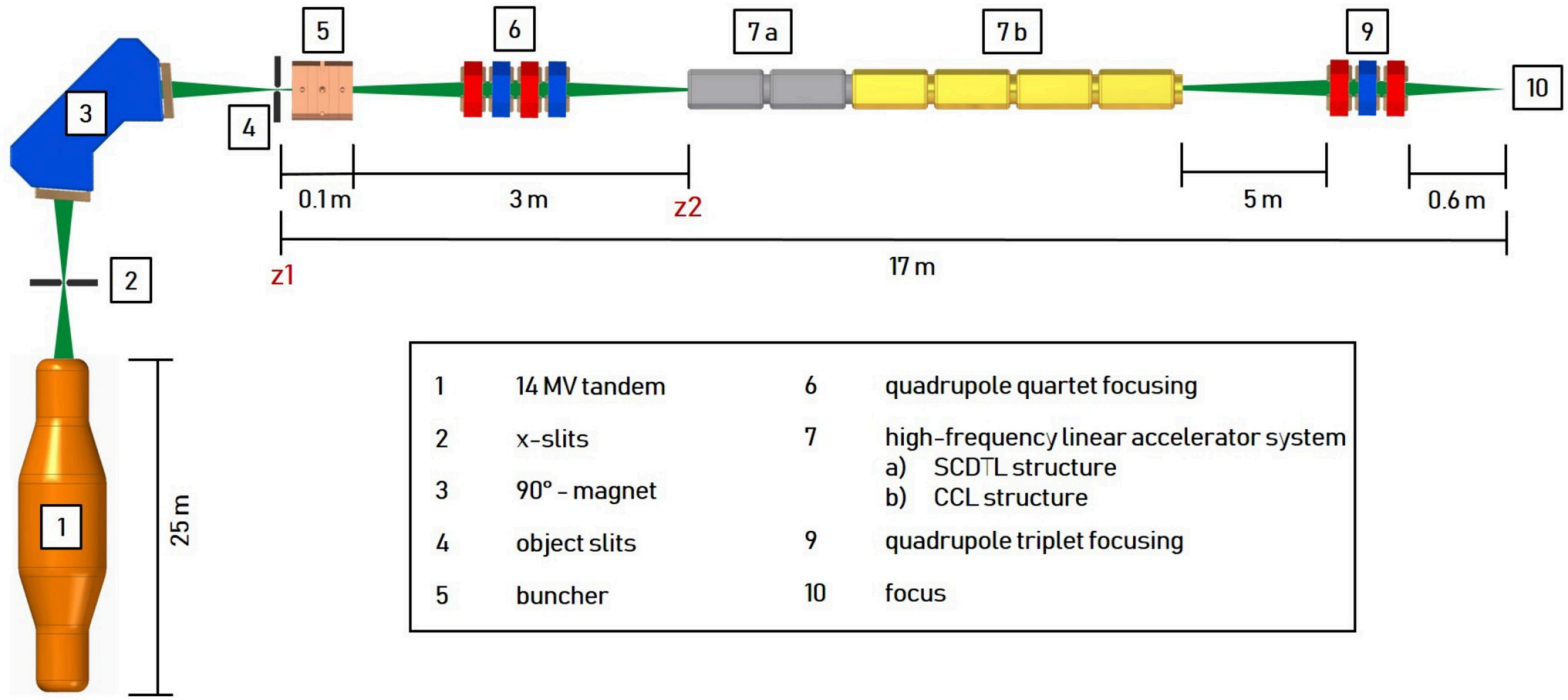
Concept of the preclinical proton minibeam irradiation facility. A 16 MeV proton beam provided by a tandem is accelerated to 70 MeV by a radio-frequency linac system and subsequently focused to 0.1 x 0.1 mm^2^ by a quadrupole triplet. The 6-dimensional phase space of the tandem beam is matched to the accepted phase space of the linac by a buncher unit and a quadrupole quartet.

A promising further development in this field is proton minibeam radiotherapy (pMBRT) [4, 5]. Submillimeter planar or pencil-like beams are applied with a beam spacing (centre-to-centre distance (ctc)) much larger than their transverse beam dimensions (at tissue entry). Typical transverse beam dimmensions range from 0.1 mm to about 1 mm and typical ctc values from 0.5 mm to a few millimeters [6, 7]. This results in a transverse dose profile characterized by a pattern of alternating dose maxima (peaks) and dose minima (valleys) and thus a high spatial fractionation of the dose in the normal tissue. A quantity frequently cited in this context is the peak-to-trough dose ratio (PVDR). A high PVDR associated with low valley doses is required to promote maintenance of normal tissue [8]. The biological mechanisms leading to this normal tissue preservation cannot yet be fully explained. However, some relevant mechanisms are summarized in [9, 10]. The lateral spread of protons, caused by small angle scattering, enlarges the individual minibeams with increasing penetration depth toward the target volume. A quasi-homogeneous irradiation and the minimization of the PVDR in the target volume is achieved by the superposition of the individual beams for a combination of transverse beam size and CTS suitable for the specific target depth. The potential of pMBRT has been successfully demonstrated in the past using various biological models as presented for example in [11–16]. First pMBRT treatment plans for treating humans have been simulated which also suggest dosimetric advantages in normal tissues compared to conventional proton therapy [17]. However, mouse ear studies suggest that side effects can be completely avoided only with proton mini-beams that have a transverse dimension of less than *σ* = 90 μm and a ctc of 1.8 mm at the tissue entrance [13]. Such beam dimensions with a sufficiently high beam current are currently only available to the pMBRT community at the Maier-Leibnitz laboratory in Munich. However, the maximum proton energy and thus the range of the protons is limited there by the terminal voltage of the 14 MV Van-de-Graaff accelerator to 28 MeV (about 6 mm proton range in water) [7]. In order to demonstrate the advantages of pMBRT with transverse beam dimensions *σ* < 90 μm at higher proton energies and thus for deeper lying tissue, a new concept for a preclinical irradiation facility (shown in Fig 1) was designed and characterised by simulations [18]. A tandem accelerator generates a 16 MeV proton beam of high brilliance that is further accelerated by a 2997.92 MHz linear post accelerator (linac) system to 70 MeV (about 40 mm proton range in water) and focused by a quadrupole triplet on the target. The linac system is based on the commercial system from AVO-ADAM called LIGHT (Linac for Image Guided Hadron Therapy) [19]. It consists of two Side Coupled Drift Tube Linac (SCDTL) structures orignially designed from ENEA for the TOP-IMPLART project [20] and 4 Coupled Cavity Linac (CCL) structures. The advantage using an existing tandem accelerator (like the one situated at the Maier Leibnitz Laboratory in Garching [21]) as injector is the reduced costs compared to the full front-end linac system [19, 20], since the radio-frequency quadrupole (RFQ) and the first modules of the linac can be omitted. Additionally, the tandem accelerator is perfectly suited to generate a bright and high intensity proton beam and can be used for other applications (e.g. materials sciences).

**Fig 2:**
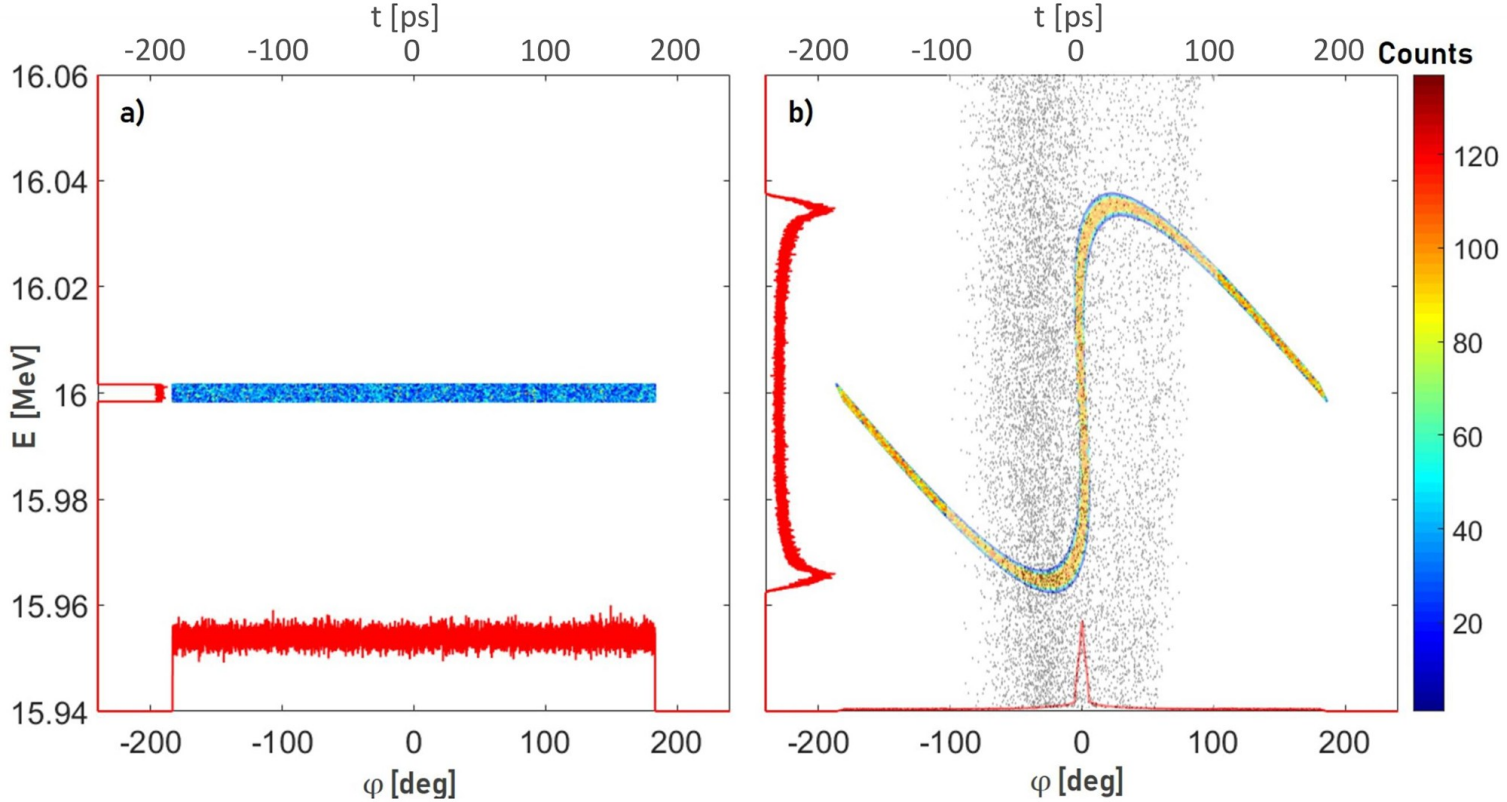
Longitudinal phase space between the tandem and linac. a) longitudinal phase space at z1 (see Fig 1) coming from the tandem (*E*_*kin*_ = 16 MeV, Δ*E*_*kin*_ = 0.01%) [18] b) longitudinal phase space in front of the linac at z2 (see Fig 1) (colored dots) and accepted phase space of the linac (grey dots). The red curves along the x- and y-axes are projections of the particle distributions.

To increase the transmission through the post-accelerator, a 2997.92 MHz buncher unit is necessary for adjusting the longitudinal phase space at position z1 coming from the tandem (Fig 2a) with the accepted phase space of the linac (grey dots in Fig 2b). The resulting time focus in front of the linac at position z2 is shown in Fig 2b (colored dots). The RF pulse characteristic of the buncher is adapted to the linac (5 μs at 200 Hz). A quadrupole quartet is used to match the transverse phase space between the tandem and linac.

**Fig 3:**
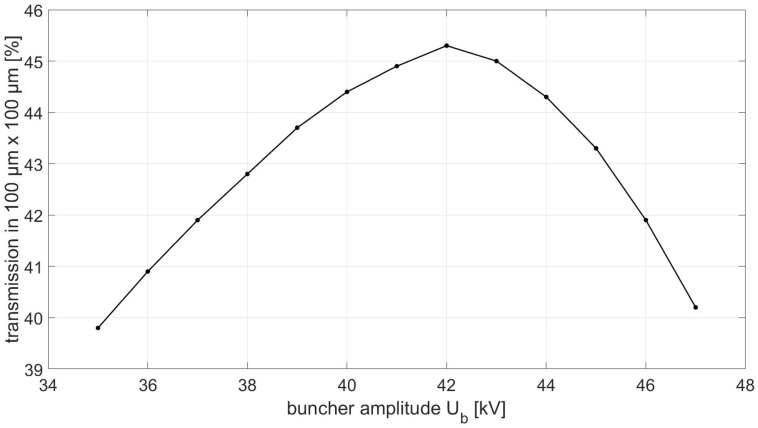
Proton transmission into an area of 0.1 x 0.1 mm^2^ at the focus (see Fig 1) as a function of buncher amplitude *U*_*b*_.

The particle tracking code TRAVEL [22] was used to optimize the buncher peak voltage (buncher amplitude) *U*_*b*_ and the field strength of the quadrupole quartet/triplet to maximize the proton transmission into an area of 0.1 x 0.1 mm^2^ at the focus. Fig 3 shows the percentage of protons transmitted from the tandem through the linac into an area of 0.1 x 0.1 mm^2^ as a function of *U*_*b*_. An optimal buncher amplitude of *U*_*b*_ = 42 kV results in a maximum proton transmission of 47%, which is a factor 3 higher than the transmission without a buncher unit [18]. For an average tandem beam current of 40 nA (5 μs at 200 Hz), this results in a beam current of *I*_*B*_ = 19 nA at the focus which is more than sufficient for preclinical experiments. The deposited dose can be changed via the irradiation time and monitored with an ionization chamber. However, the total irradiation time is a crucial parameter for preclinical experiments as well as patient comfort. Therefore, the variation of the buncher amplitude Δ*U*_*b*_ should not lead to a significant change of the dose rate in order to keep the irradiation time as short as possible. Therefore, besides the amplitude *U*_*b*_, the variance of the amplitude Δ*U*_*b*_ is a key parameter of the buncher.

This paper presents two concepts for a buncher unit which allow an efficient coupling between a tandem pre-accelerator and a linear post-accelerator as required for the described preclinical irradiation facility which offers the perspective to rapidly accelerate pMBRT research. The cavity design of the buncher units is based on the drift tube linac (DTL) concept. The design of both concepts and their performance is presented in the following.

## Materials and methods

### Buncher unit design

Since there is no commercial solution for the required 3 GHz buncher unit, the buncher concepts presented in the following had to be developed from scratch. Therefore, in addition to the design methods, the following three subsections also describe the RF cavity designs and the frequency tuning systems that comprise the buncher units.

#### Design methods

A basic initial design of the cavity geometry is performed in both cases using the SUPERFISH [23] software with the specific code MDTfish. The cavity cells are tuned to a desired resonant frequency by adjusting the drift tube gap length. Then, the key performance indicators of shunt impedance, quality factor and maximum E-field strength are evaluated [24]. Subsequently, a CAD model of the buncher-units with all non-radially symmetric to the beam-axis (z) structures like tuning rods potentially influencing the EM-fields in the cavity is developed and further analyzed using CST-Microwave [25] (hereafter referred to as CST). For the CST simulation, the material property of each component is defined (cavity: high purity copper, etc.). The CST eigenmode solver is used to calculate the electric and magnetic fields of the cavity eigenmodes. Shifts in resonance frequency (*f*_*R*_) resulting from non-symmetric structures are compensated by iterative fine-tuning of the drift tube gap length.

#### RF cavity design

The RF cavity design of both buncher units is based on a drift tube concept as it is already realized for a resonance frequency (*f*_*R*_) of 2997.92 MHz in linac structures by AVO-ADAM [26] and ENEA [20]. A multi-gap solution was favored to lower the thermal load and to operate the buncher by an existing solid-state amplifier (*P*_*amp*_ ≤1350 W). Both cavities are manufactured from OFC copper.

**Fig 4:**
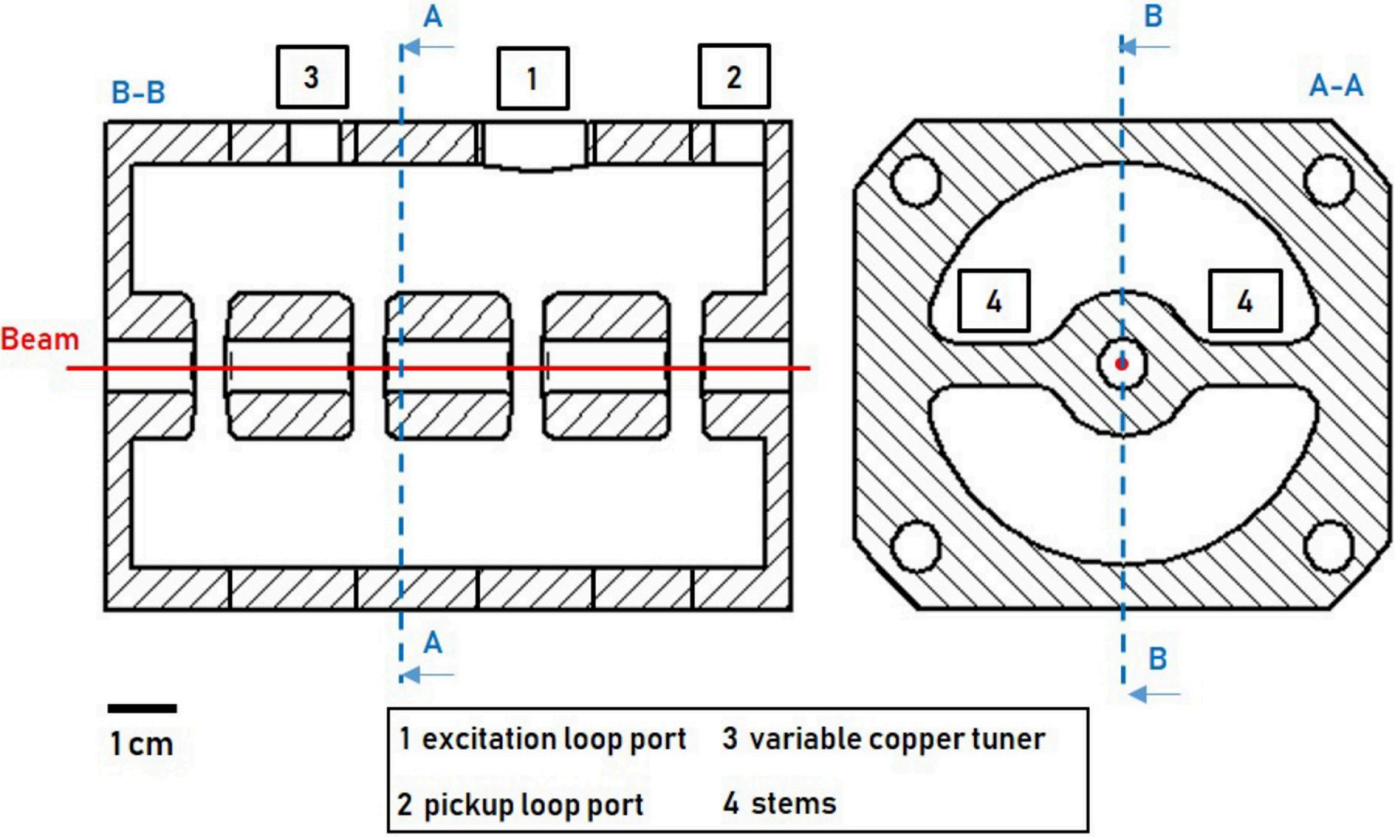
Cavity concept 1 (4-gap): Three drift tubes are attached by stems. Pickup loop, excitation loop and copper tuner have access to the cavity through ports. Red line: proton beam.

Cavity concept 1 (4-gap) was developed as part of a low-budget and study subject buncher unit. Attached to a CF-100 vacuum pot, it is placed within the beam tube, which makes cost intensive vacuum-tight joining processes (e.g. brazing) unnecessary and mounting of the buncher unit via a CF-100 crosspiece simple. At the same time, the diameter of the CF-100 flange limits the cavity geometry and the number of gaps (see Fig 6). Fig 4 shows cavity concept 1 consisting of 6 individual discs which are pressed together by screws forming 4 individual cells with one gap each. The drift tubes are supported by stems. Pickup loop, excitation loop and copper tuner have access to the cavity through ports.

**Fig 5:**
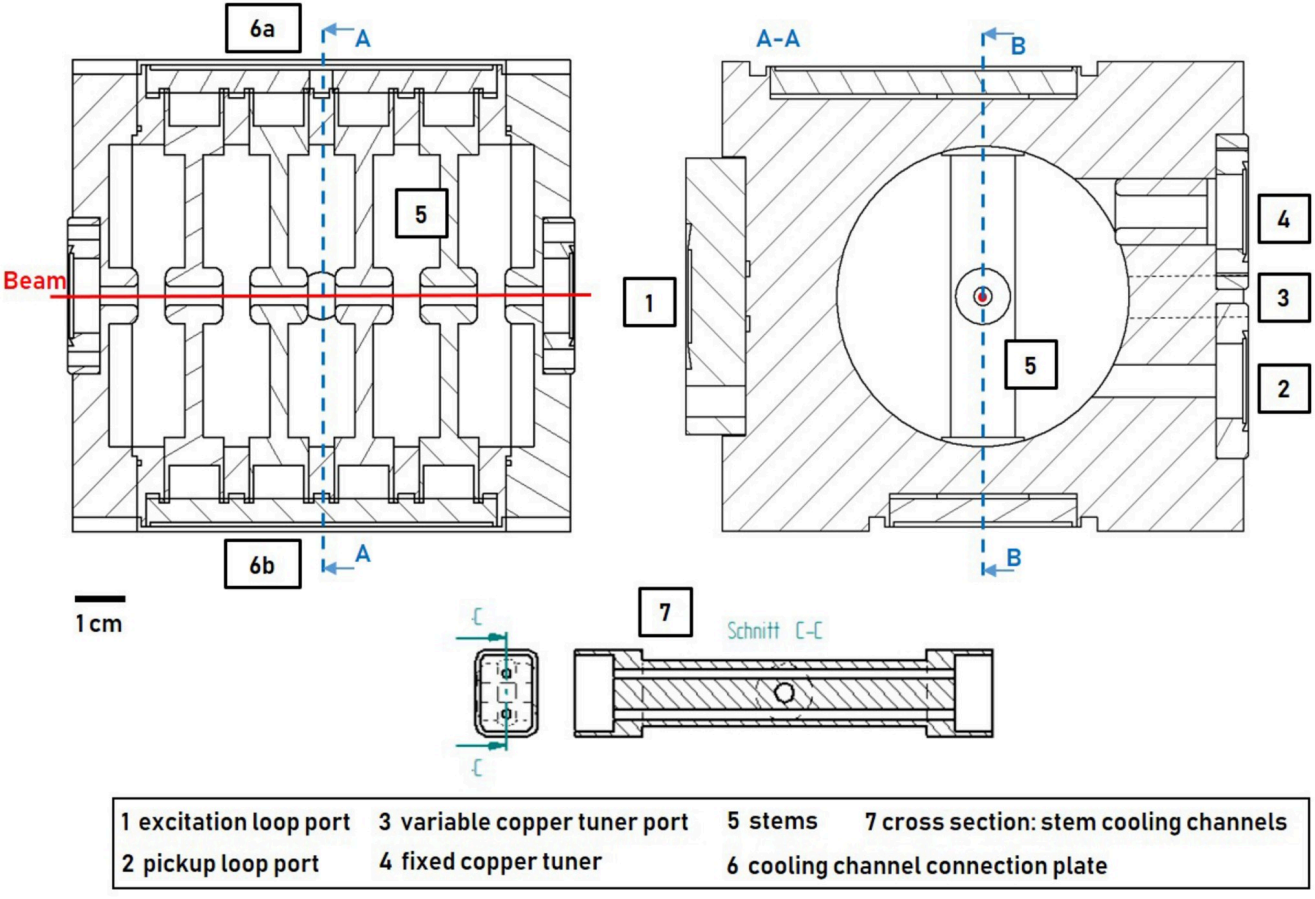
Cavity concept 2 (5-gap): Four drift tubes are attached by stems with implemented cooling channels. Pickup loop, excitation loop, fixed copper tuner and variabe tuner are depicted. Red line: proton beam.

Fig 5 shows cavity concept 2 (5-gap) which is adapted from the drift tube part of the Side Coupled Drift Tube Linac (SCDTL) as used in the TOP-IMPLART project [20]. The 5-gap cavity geometry of the TOP-IMPLART SCDTL-2 structure (insertion energy 16.5 MeV) was optimized for 16 MeV (insertion energy SCDTL 2 module of the LIGHT system [27]). The cavity was designed to reach the resonance frequency at a temperature of 42°C. Pickup and excitation loop, variable copper-tuner, and fixed copper tuner have access to the cavity through ports. The drift tube stems contain two cooling channels. All cooling channels are supplied with coolant from a reservoir which is closed by a plate (6a) in which a valve is mounted. After the coolant has passed through the stems, it is collected in a second reservoir (6b) located on the opposite side of the structure. A second valve is mounted at the second reservoir and thus a closed cooling circuit is formed when the chiller system is connected. To enable the operation of the cavity in air and to increase electrical conductivity, the cavity parts were joined by brazing. In addition, the brazing process is necessary to enable the cooling channel system.

Table 1 shows the main parameters of the basic cavity designs obtained from SUPERFISH simulations [23]. Taking into account the attenuation due to the required RF equipment, the solid state amplifier provides 950 W to drive the cavities. The input power *P*_*in*_ required for a given buncher amplitude *U*_*b*_ can be calculated from the effective shunt impedance *Z*_*TT*_ and the overall length L.

$$\begin{array}{c} P_{in}=\frac{{U_{b}}^{2}}{Z_{TT}L} \end{array}$$ (1)

**Table 1:** Parameter list of basic buncher cavities.

Parameter	4-gap cavity	5-gap cavity
Inner diameter [mm]	46.21	62.00
Drift tube diameter [mm]	17	12
Bore diameter [mm]	6	4
Overall length L [mm]	72.87	91.09
Gap length [mm]	3.74	5.55
Unloaded quality factor *Q*_0_	8813	12984
Transit-time factor	0.71	0.81
Eff. shunt impedance *Z*_*TT*_ [$\frac{M\Omega }{m}$]	35	77

Taking into account a safety margin of 25% for the simulated eff. shunt impedance, an input power of 860 W for the 4-gap concept and respectively 280 W for the 5-gap concept is required to realize *U*_*b*_ = 42 kV. Due to design restrictions of the 4-gap cavity geometry by the CF-100 vacuum pot, the eff. shunt impedance of the 5-gap concept is 2.2 times higher than that of the 4-gap concept. However, both concepts achieve the required performance in SUPERFISH simulations.

#### Frequency tuning system

**Fig 6:**
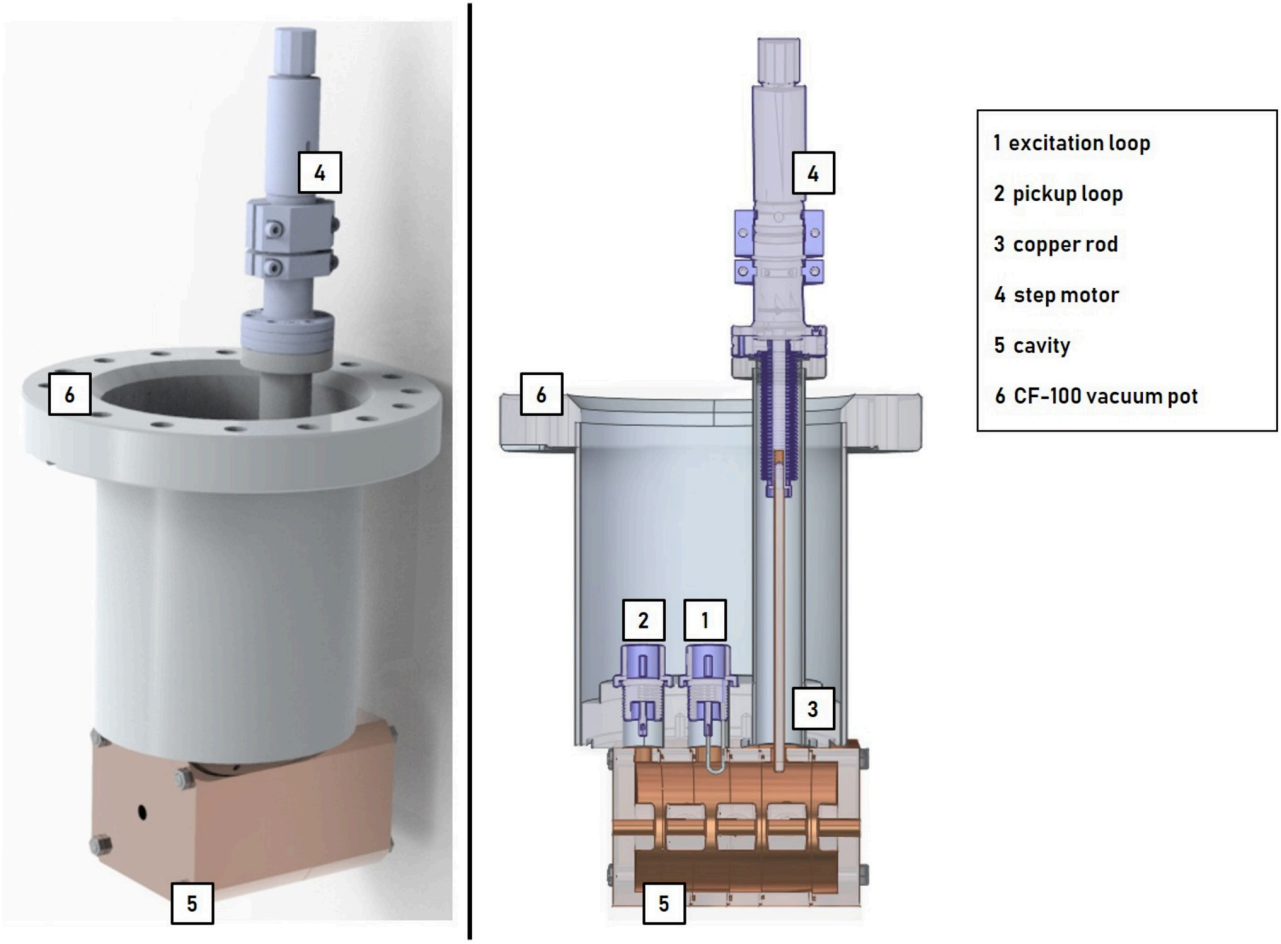
4-gap buncher unit. Mounted on a CF-100 flange, the cavity can be installed directly in the beam tube. The vacuum pot provides pickup loop, excitation loop and variable tuner access to the cavity.

**Fig 7:**
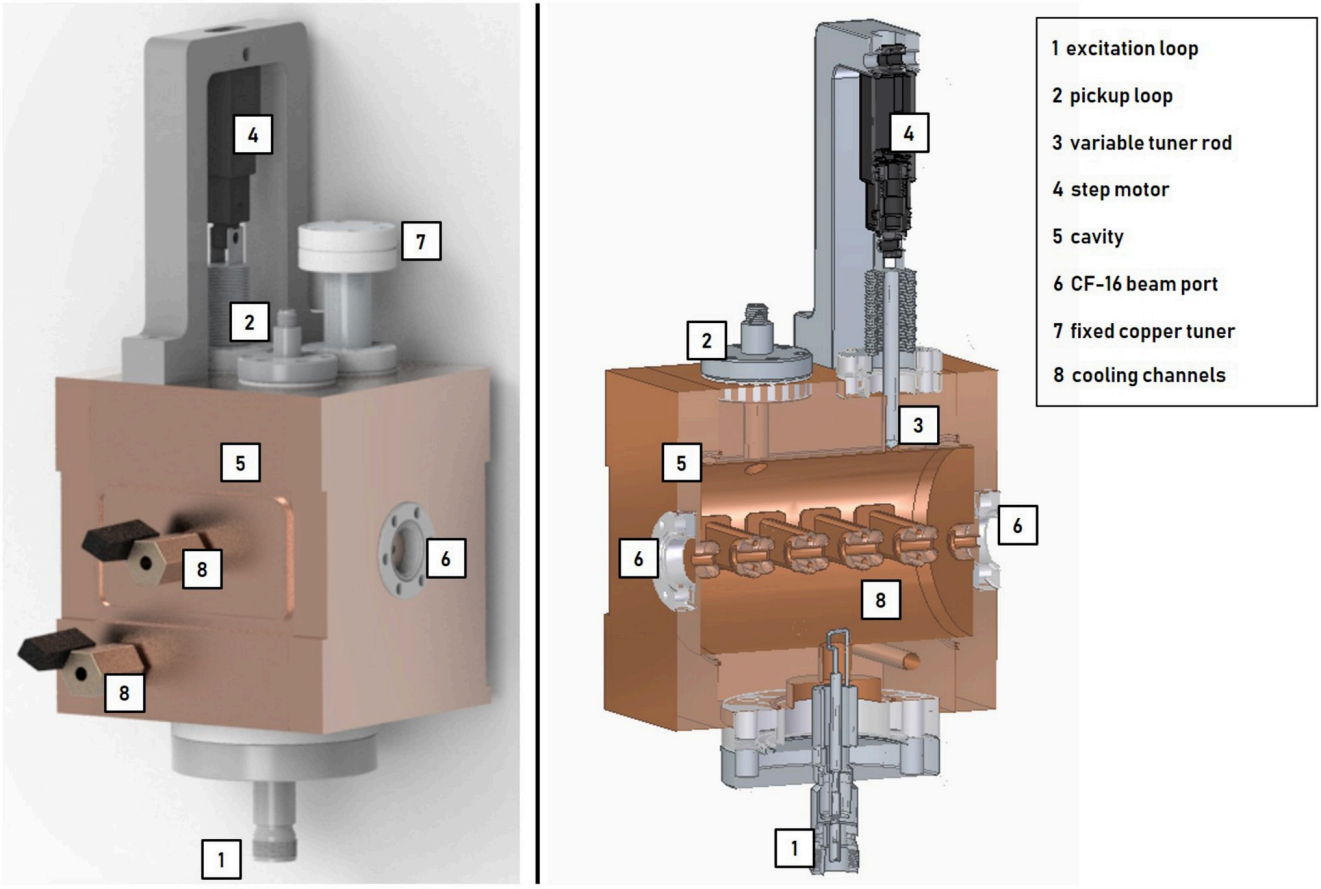
5-gap buncher unit. Connection to the beam pipe via CF-16 flanges. CF-16 flanges also provide access to the cavity for pickup loop, variable tuner and fixed copper tuner. The excitation loop is mounted on a CF-63 flange.

The frequency tuning system (FTS) of both concepts compensate for the expected thermal drift $\frac{\Delta f}{\Delta T}\approx 46\frac{\text{kHz}}{{}^{{}^{\circ}}\text{C}}$ [28] resulting from a change in surrounding temperature or input power and corrects manufacturing inaccuracies. Figs 6 and 7 show the two buncher units, 4-gap and 5-gap, including all ports, loops and tuning components. The 4-gap FTS consists of a variable tuning system (see Fig 6). A copper rod guided by a step motor can be moved in or out of the cavity to tune the cavity to *f*_*R*_. The 5-gap FTS (see Fig 7) is related to the one of the TOP-IMPLART project [29, 30]. To achieve the resonant frequency *f*_*R*_, the buncher unit must be operated at a defined temperature of 42°C, which requires the chiller system mentioned earlier. With a suitable chiller system the temperature of the SCDT structures of the TOP-IMPLART linac (consisting of 7 cavities similar to the 5-gap concept) can be kept stable at (42 ± 0.2)°C at an input power of 1.3 MW (5 μs at 200 Hz) [30, 31]. Considering that the maximum *P*_*in*_ of the buncher unit is less than 1 permille of the input power of the SCDT structure, it can be assumed that the buncher unit can be supplied by the same chiller system without losing temperature stability. Additionally, a fixed copper tuner can be used to compensate for manufacturing inaccuracies. Fast thermal changes are corrected by a small variable tuning system consisting of a copper rod and a step motor.

### Radio frequency system

**Fig 8:**
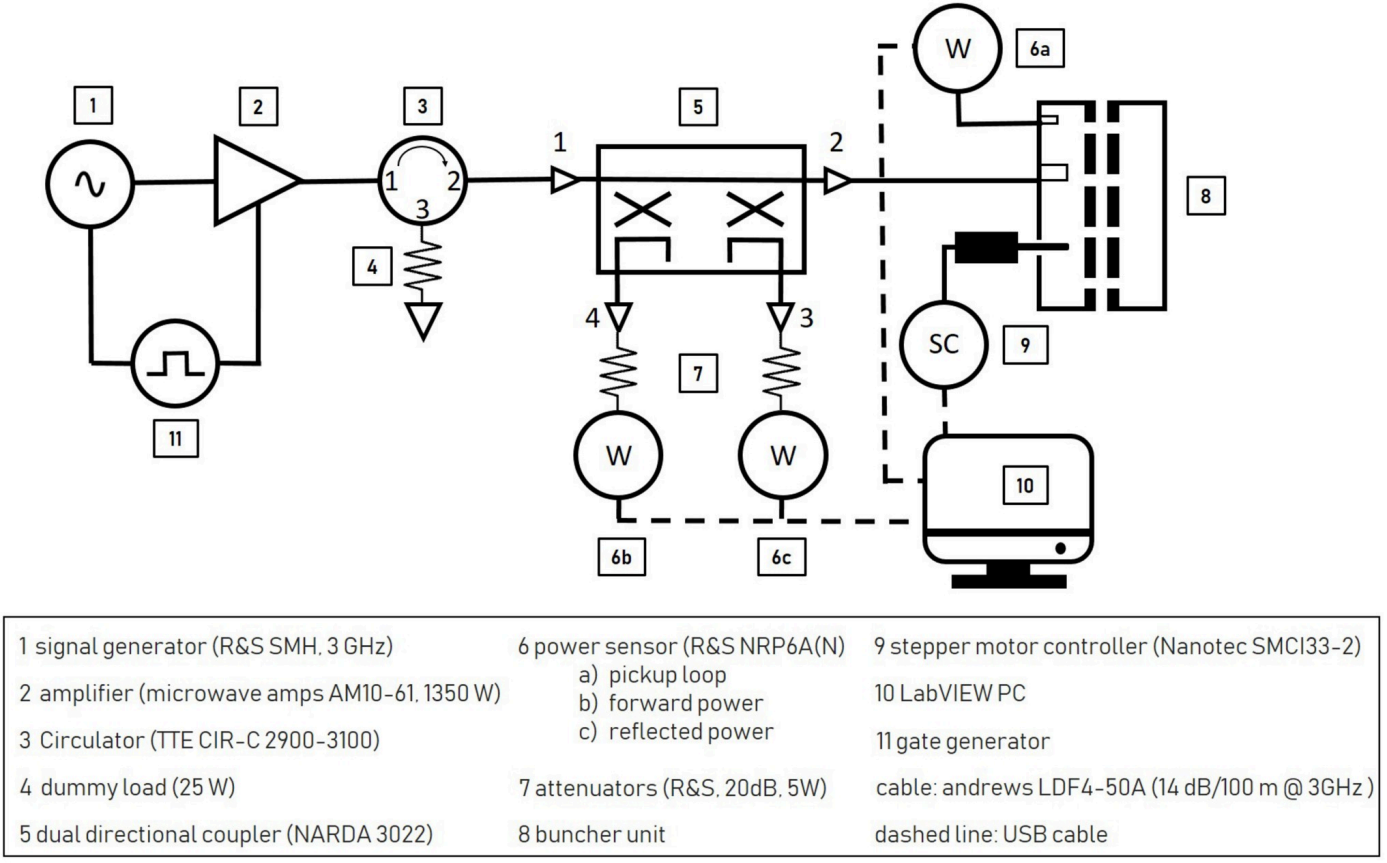
Schematic representation of frequency tuning- and RF system of the two buncher units.

**Fig 9:**
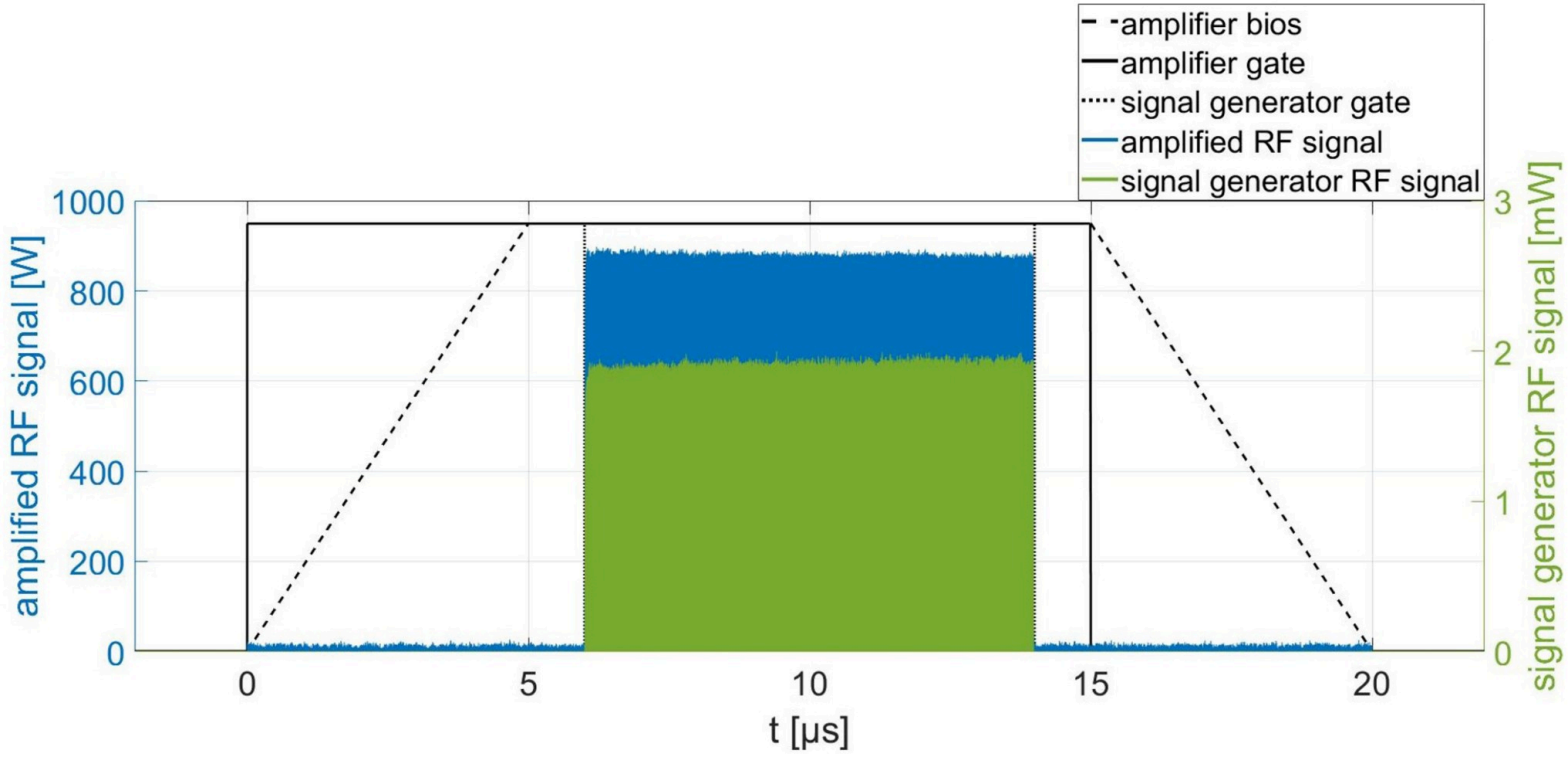
Signal characteristics of the RF system. Schematic: gate generator signal and amplifier bias. The time offset between amplified signal and generator signal is corrected for illustration purposes.

Fig 8 shows the RF system which is used to operate the buncher units. An RF signal *f*_*R*_ is provided by a signal generator and then amplified up to 1350 W by a solid state amplifier at a duty cycle of 1%. Both are synchronized by means of a gate generator. To minimize the power fluctuations of the amplifier and to protect it from reflected power, a circulator with a 25 W (cw) dummy load is following subsequently. The pickup signal Δ*P*_*pu*_ is measured by a power sensor and analyzed with LabVIEW code. To minimize power loss, all components leading to the excitation loop are connected by a cable type with a very low attenuation (14 dB/100 m at 3 GHz). Fig 9 shows the RF signal and (schematically) the gate signal of the signal generator and amplifier in detail for an RF pulse amplified to 900 W. For illustration purposes the time offset between amplified signal and generator signal was corrected. The signal generator provides an 8 μs long RF puls (green), defined by the signal generator gate (dotted line). The amplifier is ramped up (dashed line) with the beginning of the amplifier gate signal (black) and is able to provide full amplification after 5 μs until the gate signal stops. The incoming RF pulse (green) is amplified within this time window of full amplification. The output power is proportional to the amplitude of the RF pulse provided by the signal generator. The power of the amplified RF signal is stable up to 1% for a pulse length of up to 10 μs.

### Variable tuner control loop

To keep the resonance frequency *f*_*R*_ stable, both buncher concepts are equipped with a variable tuning rod. Its software control loop is based on the iterative minimization of the standing wave ratio (SWR) as shown schematically in Fig 8. Every second, two power sensors are measuring the forward and reflected power at the dual directional coupler and a LabVIEW-based control algorithm calculates the SWR. When the tuning loop is started, the variable tuner rod is inserted into the cavity until a change in the SWR is detected. Depending on whether the change shows an increase or a decrease compared to the last measured value, the direction of movement of the variable tuner is changed or maintained and is than held until the SWR increases again. This simple low budget control loop can be further optimized by analyzing the phase relationship between the input signal and the pickup signal if the variation of the buncher amplitude needs to be further reduced.

### Quality factor measurement

The unloaded quality factor *Q*_0_ (hereafter quality factor) is calculated from the loaded quality factor *Q*_*L*_. *Q*_*L*_ is, in turn, determined by the 3 dB half-width of the resonance curve of the respective buncher units, which is measured via the pickup loop. The measurements of the resonance curves were performed on the test bench where the SWR (overcoupled) was 1.05 for the 5-gap concept and 1.15 for the 4-gap concept. The input power was 10 mW (cw) in both cases. The power dissipation caused by the pick-up loop can be neglected, and thus in the case of overcoupling *Q*_0_ results to

$$\begin{array}{c} Q_{0}=Q_{L}\left(1+\text{SWR}\right) \end{array}$$ (2)

as shown in [24, 32].

### Performance measurement with a 16 MeV proton beam

**Fig 10:**
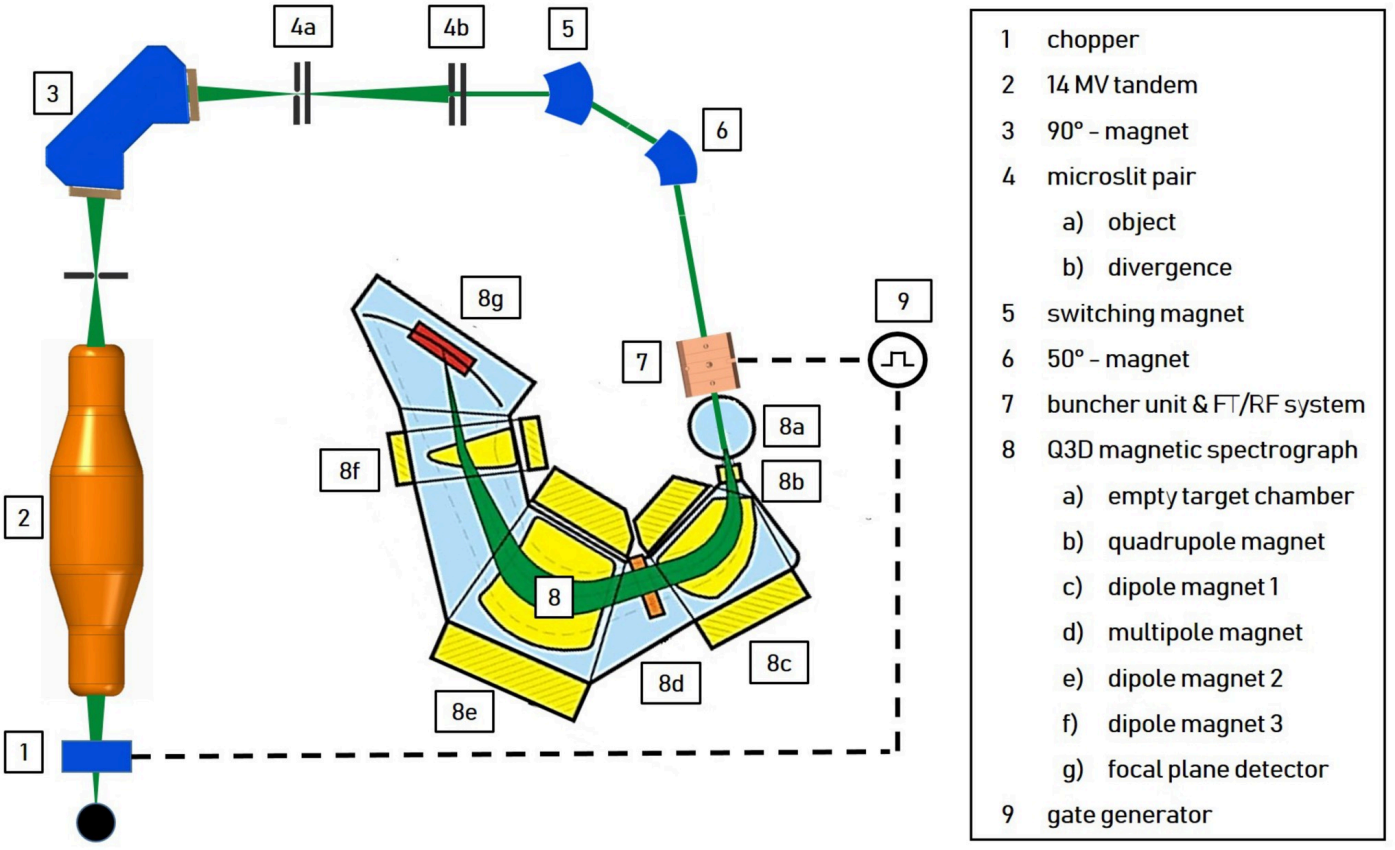
Measurement setup for performance evaluation using a tandem accelerator and a Q3D magnetic spectrograph.

The performance of the two buncher units is evaluated using a 16 MeV proton beam provided by the Munich tandem Van-de-Graaff accelerator [21] and a Q3D magnetic spectrograph [33, 34] for measuring the energy spectrum of the protons behind the buncher. The measurement setup is shown in Fig 10. During the measurements, the buncher unit under test, situated directly upstream of the Q3D, is operated with the variable tuner control loop and radio frequency system (8 μs at 200 Hz). The electric potential *U* oscillating in the cavity gaps with the resonance frequency *f*_*R*_ modulate the kinetic energy *E*_*kin*_ of the protons sinusoidally as they traverse the buncher. Depending on the buncher amplitude, this results in a characteristic energy redistribution of the protons in the longitudinal phase space (see Fig 2). The existing Q3D setup offers the possibility to measure the resulting energy histograms. Comparable to a 270° analysis magnet in combination with a segmented focal plane detector, the deflection radius of particles can be determined in relation to their energy. The detector offers a spatial resolution of 1 mm and the maximum deflection of approx. 1 m in the focal plane caused by the magnet corresponds to an energy variation of 10%. Therefore, in the case of the 16 MeV protons, the Q3D offers an excellent energy resolution of up to $\frac{\Delta E}{E}=1\cdot 10^{-4}$ and at the same time the possibility to measure the entire energy histogram at once. To protect the focal plane detector from overstressing, the beam is reduced to ≈ 300 $\frac{\text{protons}}{\text{s}}$ by a chopper unit (7 μs at 200 Hz) and microslits. A gate generator is used to synchronize the chopper system and the RF system of the buncher unit so that the proton bunches reach the buncher unit 100 ns after the start of the excitation of the cavtiy by the RF pulse (see Fig 9).

### Buncher amplitude stability measurement

The measurements to evaluate the stability of the buncher amplitude are carried out on the test bench in the RF-laboratory at a pressure of 10^−7^ mbar. The variable tuner control loop and the RF system, together with the buncher units described below, represent the test setup for these measurements. Using a dummy load, the signal generator and amplifier are brought to operating temperature before the measurements. Thus only the thermal drift caused by the input power applied to the cavity and changes in surrounding temperature need to be compensated by the control loop of the frequency tuning system. The pick-up signal *P*_*pu*_ is proportional to the buncher amplitude $U_{b}^{2}$. Thus, the variation of the pick-up signal Δ*P*_*pu*_ caused by the frequency tuning process is a measure for the stability of the buncher.

## Results and discussion

### Radio frequency system evaluation

**Fig 11:**
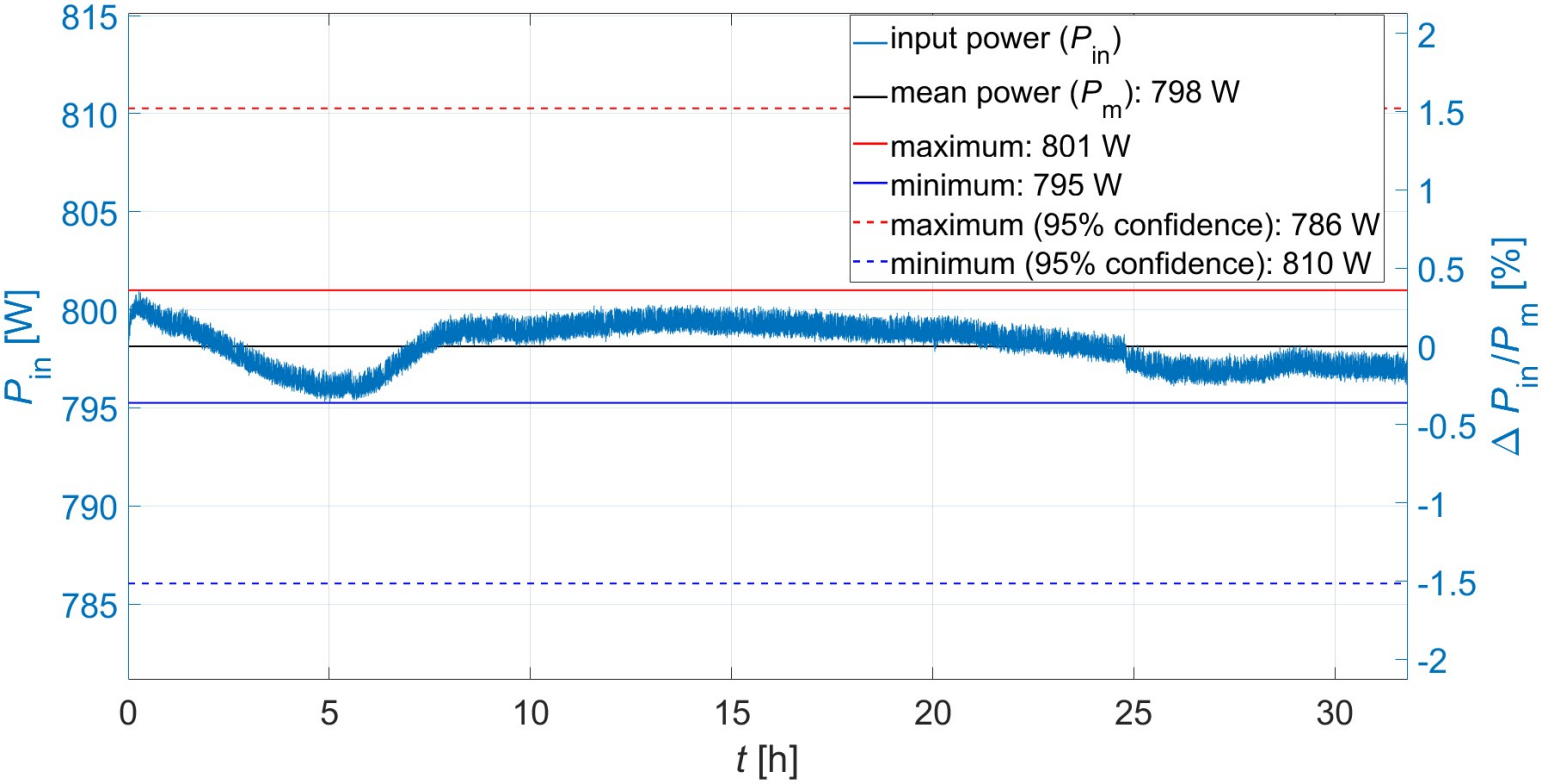
Input power stability measurement. Measuring time: 32 h.

Fig 11 shows a 32 hour long-term output signal measurement of the RF system (input signal of the buncher units) performed to estimate the stability of the RF system. The entire attenuation of the signal between amplifier output and excitation loop amounts to approx. 1.1 dB, so that 1050 W are available. The buncher unit is replaced by an RF attenuator (40 dB, 200 W) for the measurement (see Fig 8) and the output of the attenuator is connected with a power sensor (same model as in Fig 8). Each measured value of the long-term measurement results from the average of 100 single measurements taken in a measuring interval of 6 μs starting 1 μs after the RF pulse (see Fig 9). The 95% coefficient limits for the maximum and minimum input power signal are calculated from the relative measurement uncertainties specified in the sensor manual of 0.05 dB (at 3 GHz) [35]. Maximum and minimum input powers deviate from the mean input power by about ±1.5%, representing the accuracy with which the absolute value of *P*_*in*_ can be set. However, during irradiation, the input power is adjusted to achieve a maximum beam current, thus only the variation of the input power affects the variation of the beam current. In case of the 4-gap concept the maximum input power variation of about ±0.4% (at *P*_*in*_ = 795 W) results in a variation of the buncher amplitude of Δ*U*_*b*_ = ±0.1kV. The TRAVEL simulation of the presented preclinical proton minibeam irradiation facility shown in [18] were performed for the corresponding buncher amplitude *U*_*b*_ of 41.9 kV and 42.1 kV. This results in a variation of the maximum beam current of the irradiation facility (19 nA) of ±0.08%, which does not limit the planned preclinical experiments.

### Quality factor evaluation

All non-radially symmetric structures in the cavity and the change of the cavity geometry due to the movement of the variable tuner influence the electromagnetic field and consequently results in a change of the eff. shunt impedance *Z*_*TT*_. The unloaded quality factor *Q*_0_ of a cavity is proportional to its shunt impedance and therefore is a performance benchmark. *Q*_0_ was determined as described in the section Quality factor measurement and simulated with CST for both concepts for various tuner positions to evaluate the change in *Z*_*TT*_ (Table 1) calculated by SUPERFISH. For both concepts, the lowest unloaded quality factor is measured with the tuning rod extended to its maximum, i.e. the tuning rod inserted deepest into the cavity. Table 2 shows the unloaded quality factor *Q*_0_ for the expected (exp.) tuner position (arcording to the CST simulation) to reach the resonance frequency *f*_*R*_ with the required input power and for the maximum (max.) extended tuning rod.

**Table 2:** Simulated and measured unloaded quality factor *Q*_0_ for the expected (exp.) tuner position to reach *f*_*R*_ and for the maximum (max.) extended tuning rod of both buncher units.

Tuner position	*Q*_0_ measurement	*Q*_0_ CST simulation
4-gap exp.	8331	8809
4-gap max.	7845	8654
5-gap exp.	10506	10587
5-gap max.	10403	10403

The measured unloaded quality factor of the 4-gap concept for the tuner position to reach *f*_*R*_ is 6% smaller than the simulated one. This was expected since un-brazed cavities were reported to have a 7%—9% lower *Q*_0_ compared to brazed ones [36]. Besides, the surface conductivity may be reduced due to surface imperfections from machining. The measured unloaded quality factor of the 5-gap concept for the tuner position to reach *f*_*R*_ is reduced by about 1% compared to the simulated *Q*_0_. The unloaded quality factor of the 5-gap concept for the expected tuner position simulated with CST is about 19% reduced to the one simulated with SUPERFISH (Table 1). This is mainly due to the non-symmetric stems of the 5-gap concept (caused by the internal cooling channels), which cannot be included in the SUPERFISH simulation. The measured and simulated unloaded quality factor and therefore the eff. shunt impedance of both concepts correspond to the values calculated with SUPERFISH within the safety performance margin of 25% for both tuner positions. The buncher amplitude *U*_*b*_ required for the preclinical irradiation facility is achieved for both concepts in the entire tuner range.

### Performance measurement

**Fig 12:**
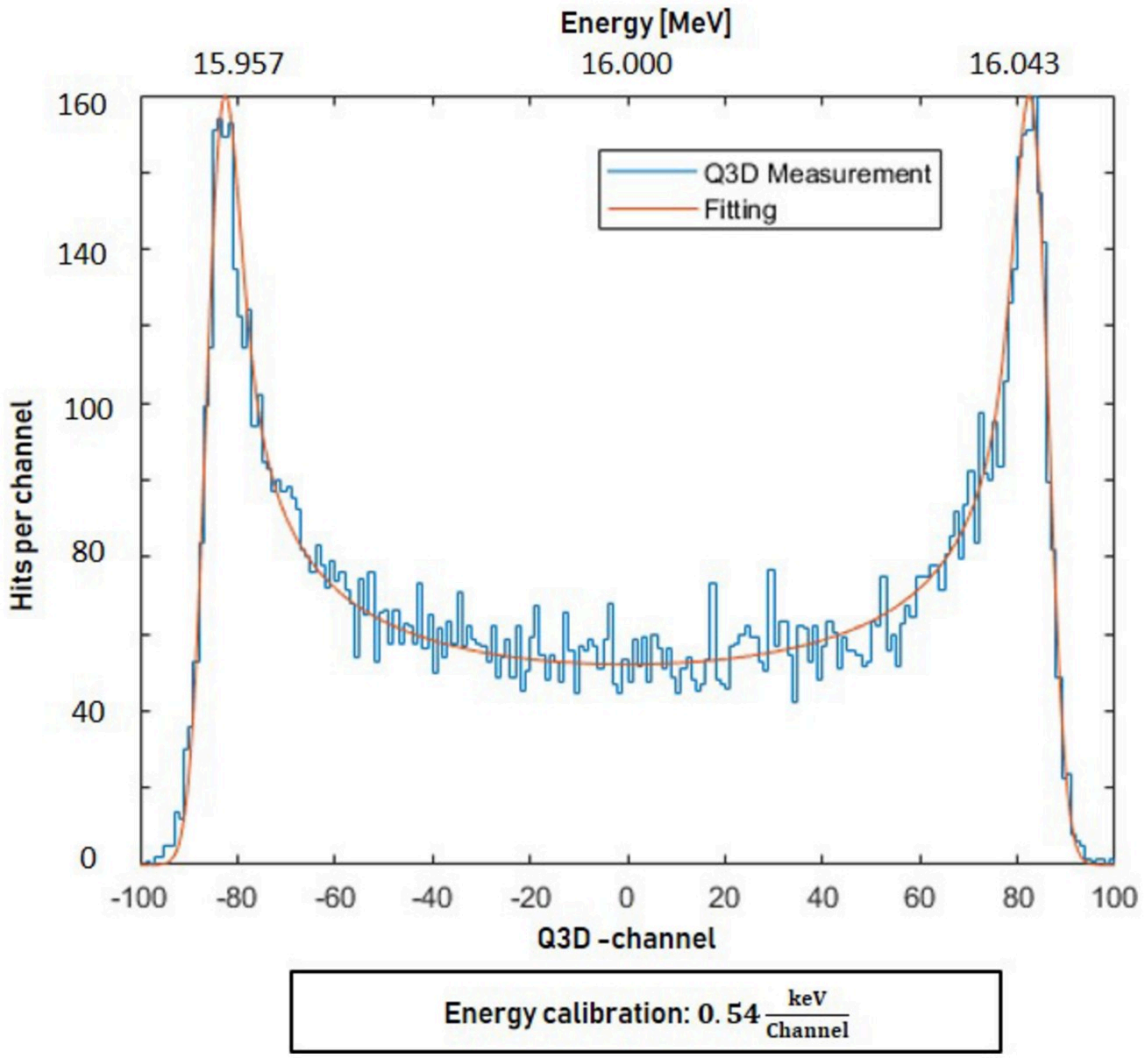
Energy distribution of the bunched protons recorded with the Q3D magnetic spectrograph setup (*P*_*in*_ = 778 W, 4-gap buncher).

The performance of the buncher units is evaluated using a 16 MeV proton beam provided by a tandem accelerator and a Q3D magnetic spectrograph. Fig 12 shows an example of an energy histogram (4-gap concept, *P*_*in*_ = 778 W) where the x-axis represents the proton energy *E*_*kin*_ (channel 0: *E*_*kin*_ = 16 MeV). The energy resolution given by the energy spread of the tandem together with the resolution of the Q3D setup was measured as a Gaussian *f*(*U*|*σ*) of *σ* = 1.7 keV ($\frac{\Delta E}{E}=1\cdot 10^{-4}$). The energy distribution function of a sinusoidal RF modulated beam without energy spread is derived in S1 Appendix for a maximum effective integrated buncher amplitude *U*_*b*_. It is transformed into a particle distribution function *g*(*U*|*U*_*b*_)

$$\begin{array}{c} g\left(U|U_{b}\right)=\frac{C}{\sqrt{U_{b}^{2}-U^{2}}} \end{array}$$ (3)

that gives the number of detected particles *N* in dependence of the acceleration voltage *U* (see also S1 Appendix). The constant *C* is proportional to the total number of measured particles. The convolution of the energy resolution with the characteristic sinusoidally modulated distribution function results in the fit function

$$\begin{array}{c} F\left(U|U_{b},\sigma \right)=g\left(U|U_{b}\right)*f\left(U|\sigma \right) \end{array}$$ (4)

that is added to the measured data (red line in Fig 12). Thus, the buncher amplitude *U*_*B*_ can be determined resulting in (42.98 ± 0.24) kV for the energy histogram shown in Fig 12. The distance between the two maxima in the Q3D focal plane is about 5.4 cm.

**Fig 13:**
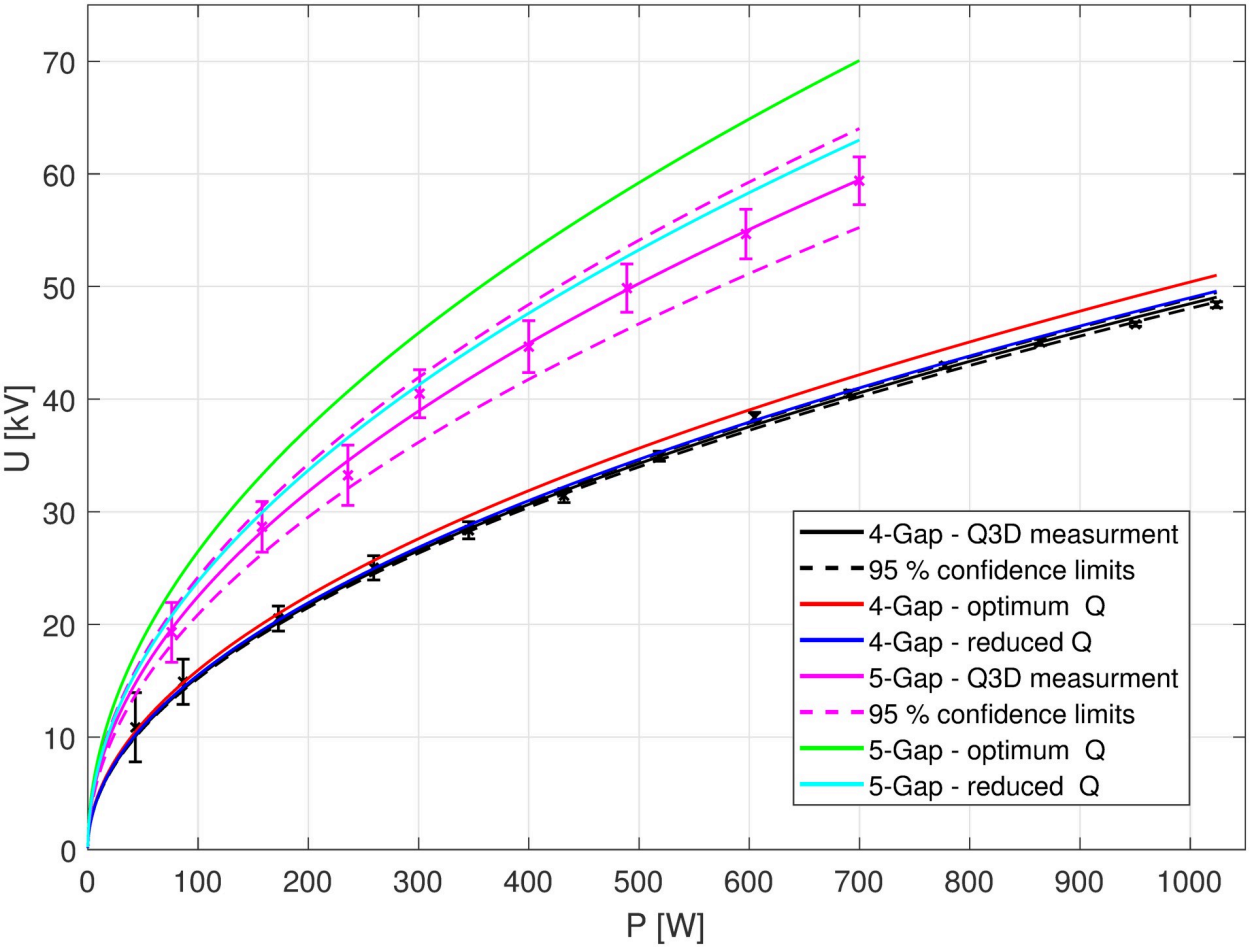
Performance of the two buncher concepts. Black and pink: Measurement series of the accelerator voltage *U*_*b*_ as a function of the input power *P*_*in*_, performed on the Q3D. Green and Red: “Best Case” scenario simulations. Light blue and dark blue: reduced measured quality *Q*_0_.

Fig 13 shows the evaluated amplitudes *U*_*b*_ as a function of *P*_*in*_ for both buncher units. The measurements (black: 4-gap concept, pink: 5-gap concept) are fitted with the function *y*(*x*) = (*a* ⋅ *x*)^1/2^ where *a* represents *Z*_*TT*_ ⋅ *L* (compare Eq 1). The dashed curves are the confidence limits (95%) for these fits. The measurement error Δ*U*_*b*_ was calculated using the *χ*^2^-squared-method to evaluate the quality of the fit. The red and green curves show the simulated performance for both concepts as calculated from the eff. shunt impedance simulated in SUPERFISH (see Table 1) using Eq 1. The performances due to the measured unloaded quality factor (see Table 2) is shown in light blue and dark blue. The performance measured on the Q3D setup correspond largely to the reduced eff. shunt impedance (resulting from the measured *Q*_0_) in both cases. The differences between simulated optimum and the measurement corresponds to the reduction of the cavity unloaded quality factor *Q*_0_ as discussed in section quality factor evaluation. The fit functions and the confidence intervals result in *Z*_*TT*_ = 32.25 ± 0.40 $\frac{\text{M}\Omega }{\text{m}}$ for the 4-gap concept and *Z*_*TT*_ = 55.49 ± 6.9 $\frac{\text{M}\Omega }{\text{m}}$ for the 5-gap concept. The required buncher amplitude *U*_*b*_ = 42 kV is achieved for an input power of 751 W for the 4-gap cavity and of 349 W for the 5-gap cavity. Compared to the simulated *Z*_*TT*_ values (see Table 1), the measured *Z*_*TT*_ values are reduced by about 8% for the 4-gap concept and 28% for the 5-gap concept. This was expected since the quality factors of the two concepts were similarly reduced compared to the SUPERFISH simulation. The reduction of the *Z*_*TT*_ values is again due to the fact that the individual parts of the 4-gap concept were not connected by soldering respectively due to the large non-radially symmetrical stems of the 5-gap concept.

### Buncher amplitude stability measurement

**Fig 14:**
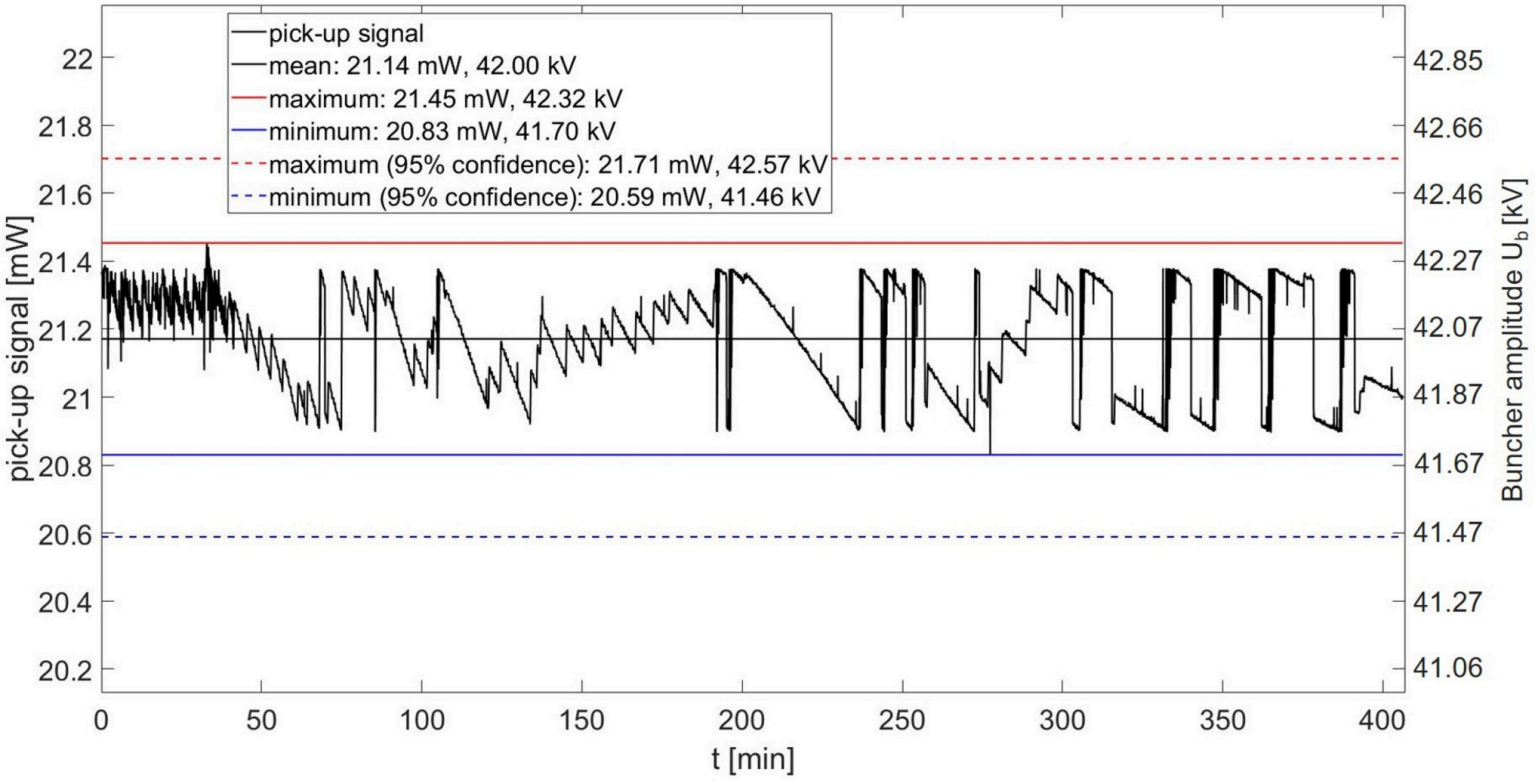
Pick-up signal measurement of the 4-gap concept with 800W (8 μs at 200 Hz) input power. Black line: Mean of the pick-up signal, red line: maximum pick-up signal and blue line minimum pick-up signal. Dashed lines: 95% coefficient interval of maximum and minimum pick-up signal.

Fig 14 shows a six-hour buncher amplitude stability measurement of the 4-gap concept at an input power of 800 W (8 μs at 200 Hz). Each value represents the average of 100 single pickup signal measurements over a measurement window of 6 μs starting 1 μs after the RF pulse as described in Fig 9. The 95% coefficient interval for the maximum and minimum pick-up signal is calculated from the relative uncertainty for power measurements of 0.05 dB (at 3 GHz) [35]. This represents the accuracy with which the absolute value of *U*_*b*_ can be set. However, during irradiation, the input power is adjusted to achieve a maximum beam current, thus only the variation of *U*_*b*_ affects the variation of the beam current. The pick-up signal shows a variation of Δ*P*_*pu*_ = ±1.5% during this time and therefore a variation of the buncher amplitude of Δ*U*_*b*_ = ±0.31kV is expected. In the case of the preclinical irradiation facility, this corresponds to a variation of the proton transmission in 0.1 x 0.1 mm^2^ (as can be seen in Fig 3) and thus a maximum variation of the beam current of less than ±0.3%. The variation of the buncher amplitude therefore has no significant effect on the dose rate. A large deviation to lower dose rates would prolong the application time of each spot and in consequence the whole treatment time. Additionally, low dose rates can lead to problems of the dose detection accuracy. Clinically used dose-monitoring systems accept typically a dose rate variation of a factor of two or more in both directions [37].

As already described in section RF cavity design, the temperature of the 5-gap concept is stabilized by a chiller system. This system is not yet available for long-term measurements, as it is part of the future linac system. The stability of the 5-gap concept is therefore based on the assumption that the system can be operated at (42 ± 0.2)°C. The shift in *f*_*R*_ due to this expected temperature drift results in $46\;\frac{\text{kHz}}{{}^{{}^{\circ}}\text{C}}\cdot 0.2{\;}^{{}^{\circ}}\text{C}=9.2\;\text{kHz}$. For the tuning rod positions at which this frequency shift is compensated (relative to the expected tuning stab position), a variation of the unloaded quality factor and thus the eff. shunt impedance of $\frac{\Delta Q_{0}}{Q_{0}}\approx \pm 0.2\%$ was measured. The *Q*_0_ values associated with these tuning rod positions were measured as described in section Materials and methods with 10 mW cw input power. Using Eq 1 and taking into account the measured stability of the RF system of $\frac{\Delta P_{in}}{P_{in}}=\pm 0.4\%$ (see Fig 11), this results in a maximum change (95% confidence interval) of the buncher amplitude of about ±0.12kV and thus a beam current stability of ≈ ±0.1%. The standing wave ratio (SWR) at the excitation loop and the quality of the vacuum show no significant changes which would indicate multipactor effects or sparking during all the test measurements (and Q3D measurements). Therefore, both buncher units can be operated long-term without loss of performance at the tested input powers. Both buncher units therefore outperform the stability requirements for a preclinical irradiation facility.

## Conclusion

Two 2997.92 MHz buncher units were developed and manufactured. The measured performance meets the expectations and the required buncher amplitude of 42 kV to provide the maximum beam current for preclinical experiments is achieved in both cases. The stability of the buncher amplitude Δ*U*_*b*_ and the resulting beam current variation of less than 0.3% in case of the 4-gap concept is more than sufficient for preclinical experiments and can be assumed to be even more stable for the temperature stabilized 5-gap concept. Therefore, both concepts outperform the requirements for the planned preclinical minibeam irradiation facility. In general, it was shown that the presented simple buncher concept, even as a low-budget version, offers a performance with which in previous simulations the beam current of a tandem pre-accelerator and linear post-accelerator combination could be increased by a factor of 3 [18]. Thus, the central, non-commercially available component of the planned preclinical proton minibeam irradiation system has been successfully developed and its performance evaluated.

## Supporting information

S1 Appendix Energy distribution function. (PDF)
