# Supplementary material for: Concept and performance evaluation of two 3 GHz buncher units optimizing the dose rate of a novel preclinical proton minibeam irradiation facility
Source: PLoS One. 2021 Oct 11;16(10):e0258477. doi: 10.1371/journal.pone.0258477 (PMC8504737; doi:10.1371/journal.pone.0258477)
Supplement: S1 Appendix — (PDF) [file pone.0258477.s001.pdf]

S1 Appendix. Energy distribution function

In the case of a particle beam with continuous particle density over time, as shown in Fig. 2a, the fraction of particles dN in a time interval dt is constant: $\frac{dN}{dt} = c$. The number of particles dN within an energy bin dE as a function of the change E in beam energy after the beam has passed through a buncher cavity is given by

$$\frac{dN}{dE} = \frac{dN}{dt \cdot \frac{\partial E}{\partial t}} = \frac{c}{\frac{\partial E}{\partial t}}. \tag{A.1}$$

For a particle beam modulated by a sinusoidal RF signal, the energy modulation $E(t)$ of a particle of charge q that passes the buncher at a time point t is described by

$$E(t) = qU_b \sin(\omega t). \tag{A.2}$$

$\omega = 2\pi f$ is the angular frequency of the RF signal feeding the buncher. U_b is the total voltage a particle can see when passing the buncher The derivative of equation A.2 is

$$\frac{\partial E}{\partial t} = qU_b\omega \cos(\omega t) = qU_b\omega\sqrt{1 - \sin(\omega t)^2}. \tag{A.3}$$

Solving equation A.2 for $\sin(\omega t)$ and substituting it into equation A.3 results in

$$\frac{\partial E}{\partial t} = qU_b\omega\sqrt{1 - \left(\frac{E(t)}{qU_b}\right)^2} = \omega\sqrt{(qU_b)^2 - E(t)^2} = \omega\sqrt{E_b^2 - E^2}. \tag{A.4}$$

Where E_b is the maximum energy imposed by the cavity to a particle traversing it. Inserting equation A.4 into equation A.1 results in the energy distribution function

$$\frac{dN}{dE} = \frac{c}{\omega\sqrt{E_b^2 - E^2}} \tag{A.5}$$

for any beam energy $E < E_b$. Using $E = Uq$ transforms equation A.5 in the particle distribution function

$$g(U|U_b) = \frac{dN}{dU} = \frac{c}{\omega\sqrt{U_b^2 - U^2}} = \frac{C}{\sqrt{U_b^2 - U^2}} \tag{A.6}$$

that gives the number of detected particles N in dependence of the acceleration voltage U for any $U < U_b$. Where U_b is the maximum acceleration voltage (buncher amplitude).
